# Epstein Barr Virus-Associated Hodgkin Lymphoma

**DOI:** 10.3390/cancers10060163

**Published:** 2018-05-25

**Authors:** Antonino Carbone, Annunziata Gloghini

**Affiliations:** 1 Department of Pathology, Centro di Riferimento Oncologico Aviano, Istituto Nazionale Tumori, IRCCS, Via F. Gallini 2, 33081 Aviano, Italy; 2 Department of Diagnostic Pathology and Laboratory Medicine, Fondazione IRCCS, Istituto Nazionale Tumori, Via G. Venezian 1, I-20133 Milano, Italy; annunziata.gloghini@istitutotumori.mi.it

**Keywords:** Hodgkin lymphoma, Epstein-Barr virus, immunosuppression, tumour microenvironment

## Abstract

Classical Hodgkin lymphoma (cHL) is a distinct clinical and pathological entity with heterogeneous genetic and virological features, with regards to Epstein–Barr virus (EBV) infection. The variable association of cHL with EBV infection is probably related to the different levels of patient immunosuppression, both locally in the tumour tissue and at the systemic level. This review paper focuses on EBV-related cHL highlighting pathogenetic and pathological features that may impact pathobiology-driven treatment for the affected patients.

## 1. Introduction

Classification of Hodgkin lymphoma (HL) evolved from histologic classifications [1,2], to the multiparameter current World Health Organization (WHO) classification [3], in which HL has been classified into classical HL (cHL), and the less common nodular lymphocyte predominant HL (NLPHL). In 1944, Jackson and Parker called NLPHL as “paragranuloma” to separate it from Hodgkin “granuloma [1].” In 1966, Lukes and Butler [2] renamed paragranuloma “lymphocytic and/or histiocytic predominance Hodgkin disease,” recognizing a nodular and a diffuse pattern. They used the term of lymphocytic and histiocytic (L&H) Reed-Sternberg (RS)-cell variant for the diagnostic tumour cell [2], now called lymphocyte predominant (LP) cell [3]. At the Rye symposium, it was decided to combine the nodular and diffuse types of the Lukes and Butler classification under the term “lymphocytic predominance Hodgkin disease” [4]. A considerable body of evidence has indicated that NLPHL exhibits features of a B-cell lymphoma, with a characteristic antigen profile and clinical behaviour [3,5]. According to its cell of origin, phenotype and type of progression to large B-cell lymphoma, NLPHL should probably be considered as a B-cell lymphoma tout court [6]. LP cells indeed are antigen-selected mutating germinal centre (GC) B cells, express CD20, CD45, BCL6 and CD40 and are surrounded by CD4^+^ and PD-1^+^ T cells in the presence of follicular dendritic cell meshworks within tumour nodules. Interestingly, multiparametric studies have shown similarities between NLPHL and T-cell or histiocyte-rich large B-cell lymphoma (THCRLBCL) [7,8]. Moreover, NLPHL may evolve to a completely diffuse T-cell–rich proliferation resembling a THCRLBCL [9,10]. The designation of these cases as THCRLBCL-like transformation of NLPHL has been recommended [10]. Classical HL is a distinct entity with heterogeneous pathological, genetic, and virological features, with regards to Epstein Barr virus (EBV) infection. Based on the morphologic characteristics of the Hodgkin Reed-Sternberg (HRS) tumour cells (lacunar cells, multinucleated giant cells, pseudosarcomatous cells) and the composition of the reactive infiltrate of tumour microenvironment, four histologic subtypes have been distinguished: lymphocyte-rich cHL (LRCHL), nodular sclerosis (NS) cHL, mixed cellularity (MC) cHL, and lymphocyte depletion (LD) cHL [2,6]. The tumour microenvironment shows a cellular composition which is characteristic for each histotype. For example, in MC cHL, microenvironmental cell types include T- and B-reactive lymphocytes, eosinophils, granulocytes, histiocytes/macrophages, plasma cells, mast cells. In addition, a great number of fibroblast-like cells and fibrosis are frequently found in NS cHL.

A fraction of patients with advanced stage disease are not cured by conventional first-line chemotherapy [5] and show either primary refractoriness to chemotherapy or early disease relapse. Recently, the treatment strategy for relapsed and refractory/relapsed HL patients included immunotherapy through the use of checkpoint inhibitors.

## 2. Epstein–Barr Virus and cHL

**Figure 1:**
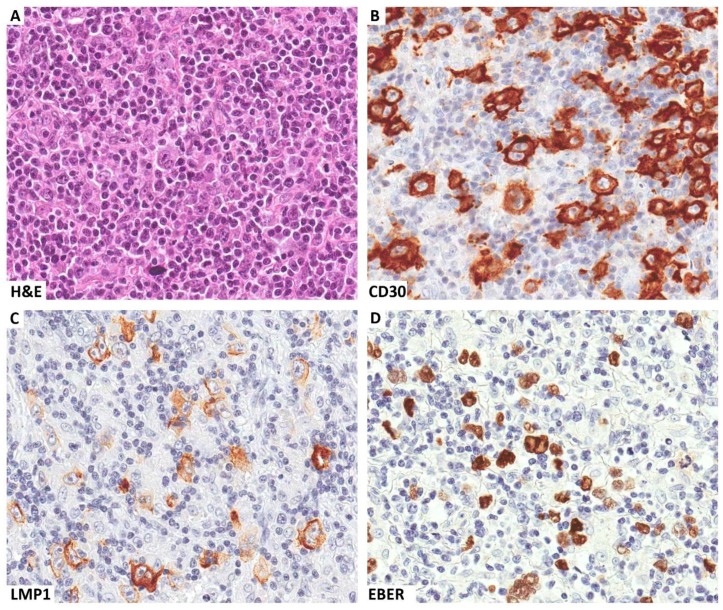
A clinical case of EBV-related Hodgkin lymphoma occurring in a patient with HIV infection. Several Reed-Sternberg cells are seen within a mixed inflammatory microenvironment (**A**). These cells express CD30 (**B**) and are EBV infected, as demonstrated by LMP1 immunostaining (**C**) and EBER in situ hybridization (**D**). Original magnification ×400 (**A**–**D**).

The immunophenotypic features of HRS cells (CD30^+^, CD40^+^, CD15^+^, IRF4/MUM1^+^) are identical in the different histologic subtypes of cHL. Conversely, the association with EBV shows marked differences: EBV is found in HRS cells preferentially in cases of MC and LD cHL, and less frequently in NS and LR cHL. Notably, the virologic characteristics of cHL vary according to the immunocompetence status of the host and cHL subtype [11] (Table 1 and Figure 1), with EBV being found in HRS cells in nearly all cases of cHL occurring in patients infected with HIV [10,12].

A pathogenic role for this herpesvirus in EBV-positive cases, probably as an early event in HL development, has been suggested [13]. The demonstration of monoclonal EBV genomes in HRS cells indicates that EBV infection occurred prior to clonal expansion. EBV-positive HRS cells express the so-called type II latency pattern including a relatively restricted set of viral genes (EBNA-1, LMP-1, and LMP-2 latent proteins, together with EBERs and BARTs RNAs) [14].

## 3. Tumour Microenvironment in EBV-Related cHL

EBV-positive cHL tissues are enriched in genes characteristic of T-cell and antiviral responses [15], suggesting that EBV has a role in influencing the tumour microenvironment. The microenvironmental cellular infiltrate of EBV-associated cHL is composed either of immune cells, including cytotoxic T lymphocytes against EBV-infected HRS cells, or of inflammatory cells supporting the growth and survival of the neoplastic clone [16,17,18].

Tumour microenvironment of EBV-associated cHLs is also characterized by a significantly higher numbers of CD68^+^, CD163^+^ macrophages than that of EBV-unrelated cHL. Coherently, overexpression of macrophage-related genes was identified in EBV-positive cHLs by gene expression profiling of whole tumour tissues [15]. Depending on availability of different microenvironmental signals, macrophages may undergo polarized activation towards two functional states: the M1 macrophages with a pro-inflammatory phenotype, having the ability to promote Th1 responses and kill tumour cells and the M2 macrophages with potent tumour-promoting activity, regulatory functions in tissue repair and remodelling and promotion of Th2 responses [19]. A main M1 polarization of macrophages infiltrating EBV-positive cHL is in keeping with a predominant Th1 microenvironment of EBV-positive cHLs [20], which is similar to, albeit less prominent of that of Th1-predominant inflammatory disorders.

LMP-1 may directly contribute to the generation of an immunosuppressive microenvironment through its ability to induce/enhance the production of immunosuppressive cytokines such as IL-6, IL-8, and IL-10 [21,22,23]. In addition, the inflammatory milieu of EBV-positive cHL is enriched in histiocytes, dendritic cells and endothelial cells.

## 4. HIV-Related Hodgkin Lymphoma

Classic HL is currently the most common type of non-AIDS-defining cancers. The pathological spectrum of HIV-related cHL differs from that of HIV-unrelated cHL. In particular, the aggressive histological subtypes of cHL—mainly MC and LD—predominate among HIV-related cHL. As observed in HIV-unrelated cHL, the so-called HRS cell is the diagnostic key for assessing this lymphoma owing to its typical morphology [24,25].

HIV-related HL shows several morphologic differences as compared with cases of the general population. Among them, the occurrence of large confluent areas of necrosis is a distinctive feature of HL occurring in HIV-infected patients and underlies the presence of a pro-inflammatory activity [26]. A “sarcomatoid” pattern, although not specific, is also observed more frequently in these cases and it has been associated with increased numbers of CD163^+^ spindle-shaped macrophages [27]. Intriguingly, a significant increase in spindle-shaped cells was observed after differentiation of monocytes into M1 compared with M2 macrophages [27]. Nevertheless, available data on the absolute numbers and functional polarization of macrophages infiltrating HIV-related HL are scarce and controversial. Unlike what it could be expected in an immune compromised setting such as HIV infection, similar numbers of HRS cells are usually found in HIV-related and HIV-unrelated cHLs [26]. This could be the result of a partially retained ability of host immune system to control the expansion of the tumour cell pool, consistently with the observation that cHL generally occurs in HIV-positive patients with a moderate level of immune deficiency, as indicated by CD4^+^ T-cell count [28,29].

In HIV-infected patients, nearly all cases of cHL are associated with EBV infection and express a type II latency. HIV-associated cHL displays biological peculiarities when compared with cHL of HIV-uninfected patients. In particular, tumour tissue is characterized by an unusually large proportion of HRS cells infected by EBV. Moreover, the fact that LMP1 is expressed in virtually all HIV-associated HL cases suggests that EBV has an aetiological role in their pathogenesis [11].

The combination of cART with better supportive therapy (such as G-CSF use and prophylaxis of major opportunistic infections) has made standard ABVD (doxorubicin, bleomycin, vinblastine, dacarbazine) and intensive chemotherapy regimens feasible also in patients with HIV-associated HL [24]. The outcome and survival of HIV-associated HL are now approaching those of HIV-uninfected patients.

## 5. Implications of Immune Evasion for Immunotherapy in EBV-Associated cHL

As for other tumours, cHL has been investigated for the expression of immunomodulatory molecules including PD-1 on T cells, and its ligands PD-L1 and PD-L2, on HRS tumour cells, which are involved in tumour cell evasion of host immune system. Classical HL is a neoplasm characterized by robust inflammatory infiltrates and heightened expression of the immunosuppressive PD-1/PD-L1 pathway. Antibodies against PD-1 have shown clinical efficacy in patients affected by cHL [30,31,32,33,34,35].

In cHL, PD-L1 expression is the result of 9p24.1 amplification and EBV infection [36,37]. Expression of PD-L1 may be up-regulated by LMP-1 in lymphoblastoid B-cell lines through activation of JAK3 and STAT5 phosphorylation or engagement of c-Jun [37]. EBV-related and EBV-unrelated cHL cases have a similar frequency of 9p24.1 gains/amplifications [38]. Moreover, EBV infection was not significantly associated the expression of PD-1, PD-L1, or PD-L2 [39]. However, EBV-related cHL had higher PD-L1 expression according to a further up-regulation of PD-L1 by viral infection [38]. According to these findings, treatments targeting PD-1 may successfully restore therapeutically immune responses against EBV-carrying HRS cells [40]. Indeed, anti-PD-1 therapy can be really effective in patients with refractory cHL. Improved treatment options, however, are needed for CHLs which are resistant to anti-PD-1 or relapse after this form of immunotherapy. A deeper understanding of immunologic factors in the cHL microenvironment might support the design of more effective treatment combinations based on anti-PD-1. In addition, because the EBV residing in CHL tumours is strongly immunogenic, characteristics of the tumour immune microenvironment in EBV-unrelated cHL would be distinct from EBV-related cHL, with specific implications for designing combination treatment regimens. A recent study has shown that the microenvironmental Th profiles are strikingly different, with EBV-related cHL demonstrating a T helper 1 (Th1) profile, whereas EBV-unrelated cHL has a Th17 profile. These results can address potential correlations of tumour response or resistance with EBV status, and with expression of a pathogenic Th17 profile, in cHL patients receiving anti-PD-1 monotherapy [41].

## 6. Conclusions

The virologic characteristics of cHL vary according to immunocompetence status of the host and cHL subtype (Table 1), while paediatric HLs are often EBV-positive [42]. Different pathogenic pathways are variably triggered by interactions of HRS cells with critical microenvironmental components and concomitant EBV viral infection. In EBV-associated cHL, LMP-1 viral oncoprotein may directly contribute to generation of an immunosuppressive microenvironment. The presence of enhanced immunosuppressive features, with high numbers of M2 macrophages and elevated expression levels of PD-L1 should make EBV-related cHL patients more susceptible to checkpoint blockade [18].

## Figures and Tables

**Table 1:** EBV infection in Hodgkin lymphoma according to the immunocompetence status of the host.

Host	Hodgkin Lymphoma	EBV Infection
Without known immunosuppression	NLPHL	Usually absent
	cHL, nodular sclerosis	Variably present
	cHL, mixed cellularity	Usually present
	Rare cHL subtypes	Variably present
With acquired Immunodeficiency	HIV-associated cHL	Present
	Post-transplant, cHL type PTLD	Present
	Iatrogenic (methotrexate)	Variably present

cHL, classical Hodgkin lymphoma; NLPHL, nodular lymphocyte predominant Hodgkin lymphoma; PTLD, post-transplant lymphoproliferative disorder.
